# Integrating early stimulation and play at scale: study protocol for “MAHAY Mikolo”, a multi-arm cluster-randomized controlled trial

**DOI:** 10.1186/s12889-022-12640-z

**Published:** 2022-02-09

**Authors:** Emanuela Galasso, Lisy Ratsifandrihamanana, Ann M. Weber, Caitlin Hemlock, Mathilde Col, Maria Dieci, Norotiana Rakotomalala, Valerie Rambeloson, Lia C. H. Fernald

**Affiliations:** 1 Development Research Group, The World Bank, DC Washington, USA; 2 Centre Médico-Educatif ‘Les Orchidées Blanches’, Antananarivo, Madagascar; 3 School of Public Health, University of Nevada Reno, Reno, NV USA; 4 School of Public Health, University of California Berkeley, Berkeley, CA USA; 5 Programme National de La Nutrition Communautaire, Antananarivo, Madagascar; 6 World Bank Office, World Bank, Antananarivo, Madagascar

**Keywords:** Early Childhood Development, Early Stimulation, Implementation Science, Time-use, Program Take-up, Play Materials

## Abstract

**Background:**

Hundreds of millions of children living in poverty worldwide are not reaching their full, developmental potential. Programs to promote nurturing and responsive caregiving, such as those in which community health workers (CHWs) conduct home visits to support optimal early childhood development (ECD), have been effective in small trials, but have not achieved similar success at scale. This study will explore two approaches to scale-up: converting a home-visiting model to a group-based model; and integrating the ECD curriculum into an existing government program. The objectives of the study are to: 1) Measure how the integration of ECD activities affects time and task allocation of CHWs and CHW psychosocial wellbeing; 2) Examine how the integration of ECD activities affects caregiver-child dyad participation in standard health and nutrition activities; and 3) Explore how the availability of age-appropriate play materials at home affects caregiver-child dyad participation rates in a group-based ECD program.

**Methods:**

We will randomize 75 communities in rural Madagascar into three arms: 1) [C], which is the status quo (community-based health and nutrition program); 2) [T], which is C + ECD group sessions [T]; and 3) [T +], which is T with the addition of an enhanced play materials package for home use. All children between 6–30 months old at the time of the intervention launch will be eligible to participate in group activities. The intervention will last 12 months and is comprised of fortnightly group sessions in which the CHWs provide caregiver-child dyads with information relating to ECD; CHWs will also include structured time for caregivers to practice the play and child stimulation activities they have learned. We will administer monthly surveys to measure CHW time use and task allocation, and we will leverage administrative data to measure caregiver-child dyad participation in the group sessions.

**Discussion:**

The results from the trial will provide the evidence base required to implement an integrated package of nutrition, health and ECD promotion activities at scale in Madagascar, and findings may be relevant in other low-income countries.

**Trial registration:**

This trial is registered on the AEA Social Science Registry (AEARCTR-0004704) on November 15, 2019 and on ClinicalTrials.gov (NCT05129696) on November 22, 2021.

## Background

Hundreds of millions of children around the world live in poverty, and as a result, are at risk of not reaching their developmental potential. Living in poverty increases exposure to multiple risk factors, such as poor nutrition, unstimulating home environments, inadequate water and sanitation, and environmental hazards, all of which can then lead to lower early childhood development (ECD) outcomes and educational attainment early in life, as well as worse labor market outcomes later in life [1–3]. Investments to promote developmental outcomes in early childhood – often in the form of parenting support/education and nutrition supplementation – can put children on a higher lifetime income trajectory, and can potentially crowd-out negative effects of early adverse events [1, 4]. A recent review of the effectiveness of parenting programs showed that, on average, participation in parenting programs resulted in improvements in child development outcomes [5]. Specifically, there were benefits for measures of cognitive development (standardized mean difference (SMD) 0.36, 95% CI 0·22–0·49, 19 studies), motor development (SMD 0.13, 95% CI 0·07–0·19, 9 studies), and socio-emotional development (standardized mean difference [SMD] 0·35, 95% CI 0·14–0·56, 13 studies). However, programs that have been effective in small program settings have not consistently achieved similar successes at scale.

A recent study in Madagascar (MAHAY) investigated the impact of an adaptation of a parenting program originating in Jamaica, the *Reach Up and Learn* program [6]. The program included fortnightly home visits by a dedicated community health worker (CHW) focusing on early childhood development (ECD) outcomes. Although the MAHAY program was carefully implemented and rigorously supervised, it failed to demonstrate significant effects on child outcomes. Researchers hypothesized that one of the reasons the intervention was not as effective as anticipated was that CHWs had to visit a large group of eligible children spread over a wide geographic area, resulting in long travel times and threats to personal safety. Another possible reason for the small effect sizes of the program on child development was the limited engagement by caregivers, possibly from lack of time or interest in topics covered. Furthermore, the caregivers may not have been able to act on suggestions made by CHWs because they did not have time or resources, or because they lacked materials, such as toys, and thus could not engage in the CHW suggestions regarding play.

A group adaptation of the previously tested MAHAY home visits was designed to address the issues that emerged in the MAHAY study. Over the course of a year, the adaptation of the group-based program was extensively pre-piloted in two communities. The revised, group-based program (MAHAY Mikolo, *“know how to take care”*) targets existing implementation issues that arose in the home-visit model by increasing coverage while reducing the amount of travel time spent by the CHWs given the greater efficiency possible with groups compared with one-on-one visits. The MAHAY Mikolo program incorporates behavioral nudges into the ECD group sessions to improve parental skills, promote peer support and encourage sustained caregiver-child play in between the fortnightly meetings. A subgroup of program communities will also receive boxes of age-appropriate toys and books to promote sustained play between group sessions and for daily practice at home. The group-based program has the potential to be integrated into Madagascar’s ongoing national reproductive, maternal and child health and nutrition program and rolled out gradually by the National Nutrition office (ONN) in Madagascar. Yet, there are important knowledge gaps about the potential costs or benefits of such an integration before scaling it up. A recent special issue in the Annals NY Academy of Science highlighted the importance of program implementation, and understanding issues such as fidelity, coverage, dosage, quality of delivery, and participants’ acceptance and enactment of program content, for program success [7]. The proposed evaluation builds from these lessons and assesses the feasibility of a group-based program by addressing key implementation research and policy questions.

The overall objective of the study is to examine the effects of integrating ECD group sessions into the existing, at-scale, community health and nutrition programs administered by the government in Madagascar by performing a process evaluation. The specific objectives of the study are to test how integrating ECD group activities affects the time and task allocation of CHWs and if adding these new activities may change the allocation of times towards health and nutrition activities, whether participation rates in health and nutrition activities are affected in communities where the ECD activities are offered, and if there is an additional benefit to providing age-appropriate toys and play materials for use at home on the take-up of ECD activities. To achieve these objectives, we will use a cluster randomized controlled trial design with three arms: a control group, with the status quo community-based health and nutrition program; a treatment arm with fortnightly group ECD sessions, based on Reach Up and Learn curriculum and supportive behavioral nudges, added to the status quo; and an enhanced treatment arm, where the intervention in the treatment arm is layered with the provision of individual toy boxes including age-appropriate toys. Detailed costing information on all activities using the ingredient methods will collected as part of the trial [8].

### Research questions

The primary questions we aim to address through this research are:

#### CHW time and task allocation

•Primary Question 1: How does the integration of ECD activities into existing health and nutrition responsibilities affect the time use and task allocation of CHWs?
•Secondary Question 1: How does the integration of ECD activities affect measures of CHW psychosocial wellbeing?

The role of CHWs has expanded considerably over the past 50 years, particularly in low and middle income countries [9, 10]. CHWs are frontline workers tasked with bringing a wide variety of public health services to their communities, and crucially bridging the gap between remote communities and the formal health care system in a hub and spokes model. A large and growing body of evidence documents the positive effects of CHW-run programs on population health [9]. Understanding the impact of the demands of the CHW role on the health and wellbeing of the workers is an important dimension of research that has received limited attention.

CHWs are often time constrained, and thus it is crucial to understand how the addition of new ECD responsibilities will affect the time spent by CHWs on regular tasks relating to health and nutrition programming. There may be economies of scope through joint delivery. Specifically, by relying on an existing infrastructure and using the same CHWs, there may be substantial cost savings to having the two sets of program components delivered separately. On the other hand, these components may interfere or crowd each other out if the time, mental space and attention of the CHWs limits the implementation of multiple tasks. Crowd-out has been documented in the context of school meals and school-based nutrition interventions in other low- and middle-income country settings [11, 12].

The evidence on the positive effects of CHWs on health knowledge and outcomes in their target communities has been shown in a variety of contexts [9], but there has also been evidence of more troubling outcomes such as service delivery gaps [13, 14], infrequent home visits [15], and insufficient time spent on core health promotion activities [16, 17]. These intermediate outcomes that relate to how program demands impact the quality and composition of the time spent working by CHWs are crucial to measure how CHW programs impact downstream health knowledge and outcomes.

One important pathway that affects CHWs’ ability to effectively delivery primary health services to their communities is CHW workload. Existing literature on CHW time use has found evidence of workload strain. In one study in India, high administrative burdens resulted in CHWs spending time on administrative reporting tasks such as filling out paper registries instead of direct service provision [12]. Other papers in different settings have found that a source of strain for many CHWs is a lack of compensation commensurate with the demands of their jobs [18, 19]. This study will add to the evidence base of how increasing the scope of CHW workload affects their time and task allocation, not only on the different tasks that are related to the overall package of activities, but also the potential reallocation away from the CHW’s income generating activities or household caregiving or family chores.

Our secondary objective is to measure how expanding the CHW workload through the addition of ECD group sessions affects CHWs psychosocial wellbeing over the course of the program implementation. We have a unique setting in that we will repeatedly measure the changes in time demands on CHWs through the integration of ECD group sessions over a 12-month period. Measuring changes in CHW stress and psychosocial wellbeing over this period will provide some of the first quantitative evidence for the broader implications of a change in job responsibilities on health worker wellbeing.

#### Caregiver-child dyad participation

•*Primary Question 2: How does the integration of ECD activities affect participation by caregiver-child dyads in all activities led by CHWs?*
•*Secondary Question 2: How is the treatment effect on caregiver-child dyad participation in health and nutrition activities moderated by key household and community characteristics?*

In addition to understanding time use among CHWs, this study also seeks to examine caregiver behavior and how the addition of ECD activities may affect participation of caregiver-child dyads. Attendance at the ECD sessions is likely to be time consuming, competing with caregivers’ productive activities and housework/caregiving duties, thereby negatively affecting take-up/participation of nutrition health activities. Conversely, the introduction of ECD group sessions may increase the caregivers’ potential interest and knowledge, because of its novelty and supportive nudges. Consequently, one could expect increased take-up of nutrition/health activities for existing caregiver-child dyads or new registration for health and nutrition programs of caregiver-child dyads who are in the community. The main outcome of interest will be an index of participation in the standard nutrition and maternal/child health activities. We will also examine how participation in the CHW-led activities may be affected by key household-level (e.g. maternal education), and community-level (e.g. remoteness) characteristics. A previous study found that there was some suggestive effects of heterogeneity of ECD outcomes by child age and maternal education [6]; measuring intermediate outcomes could be helpful in further understanding pathways in ECD.

#### Age-appropriate play materials

*• Primary Question 3: How does the availability of age-appropriate materials/activities for home-based play affect caregiver-child dyad participation in ECD activities?*

Toys, books and other age-appropriate play materials are crucial for young children to use in play because of the ways that these items promote engagement and promote learning. In addition to clear benefits of these items to promote children’s development, toys and books are also critical to sustain mothers’ interest in ECD activities by creating opportunities for home-based play. A recent meta-analysis including 13 RCTs showed medium-to-large benefits of stimulation interventions for improving the home caregiving environment, which includes having materials such as books and toys for children to play with [20]. A key component of our behavioral theory of change (the Integrated Behavioral Model) goes beyond motivation and knowledge and focuses on the idea that environmental constraints, such as a home without appropriate play materials, should not hinder the application of the new knowledge [21]. Another pillar of our design is that the behavior (developmentally-appropriate play and stimulation) should be salient to the individual, which is the reason we are focusing on the provision of toys, books and materials for participants to take home as a way to enhance engagement. Finally, we intend for the experience performing the behavior to become habitual so that true behavior change can occur and aim to promote habits with a combination of a toy box that is provided at home to each target child, as well as a rotating set of developmentally appropriate toys and books that participants can take home and use in between group sessions.

## Design

### Study setting

**Fig. 1:**
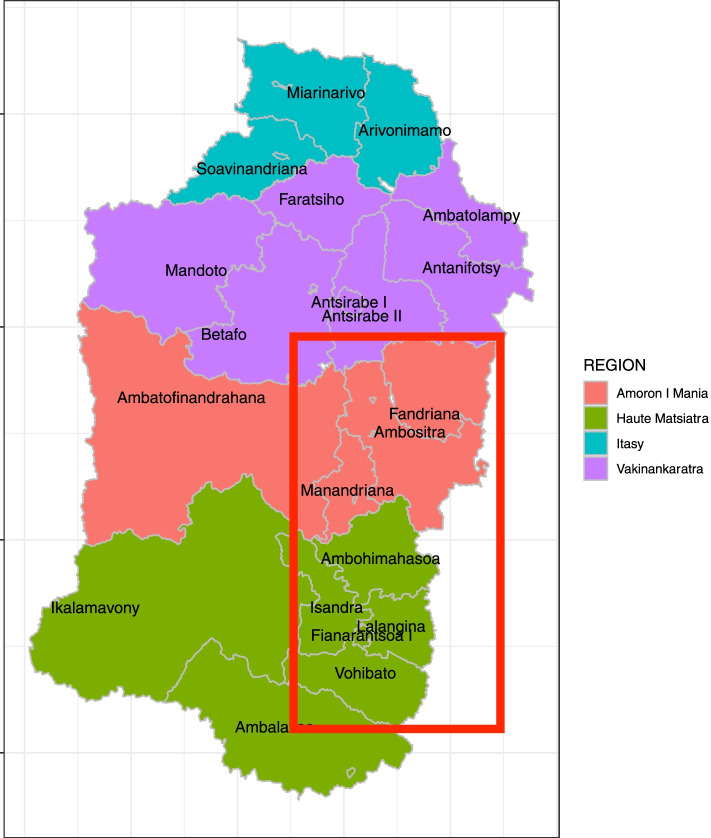
Map of districts in Madagascar. Note: Study will take place in the regions of Haute Matsiatra and Amoron i’Mania, excluding the districts of Ikalamavony and Ambatofinandrahana. The map was created by the authors using R (source of geodata shapefiles, administrative level 4: United Nations Office for the Coordination of Humanitarian Affairs (OCHA)).

Our study will take place in 75 communities in two regions (Amoron’i Mania and Haute Matsiatra) out of four regions currently covered by the existing community-based health and nutrition program (Fig. 1). The region of Itasy was excluded from our sample frame due to an ongoing LNS supplementation program and Vakinankaratra was excluded due to weak institutional capacity in that region. The study population is composed of CHWs serving these communities at the time of randomization, and caregivers with children between 6 and 30 months of age.

### Eligibility criteria

**Fig. 2:**
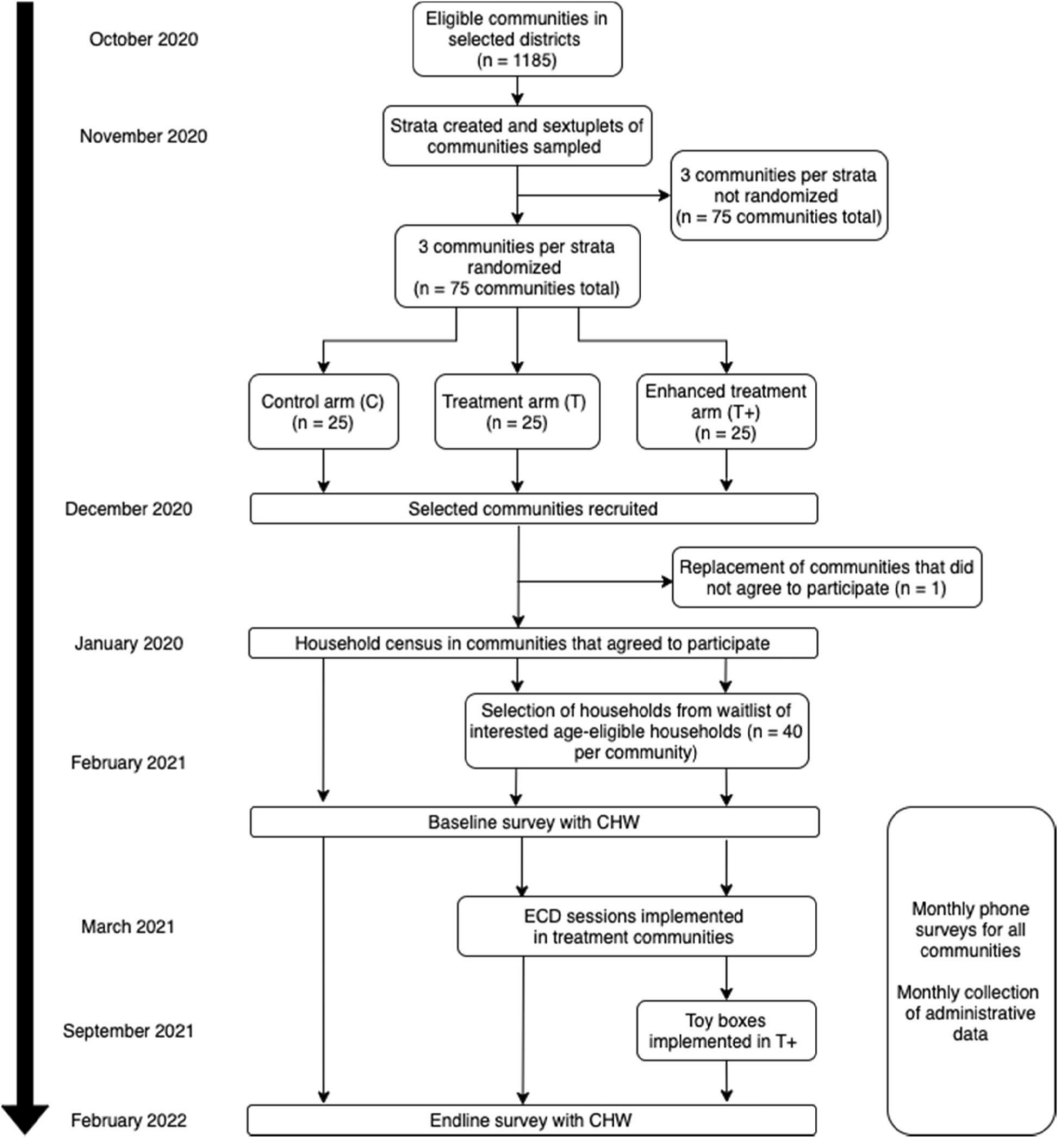
Flow of study implementation

Our sampling frame draws from the universe of communities participating in the national health and nutrition program as of September 2020 in the regions of Amoron’i Mania and Haute Matsiatra. Districts with extreme problems of accessibility (an issue for frequent monitoring) were ineligible and excluded (Fig. 2). From this universe of 1,185 potential communities in eight districts, the following inclusion criteria were used to select communities, for operational feasibility:

•No other ECD programs present in the community or neighboring communities;
•At least 40 children monitored at the nutrition center in the target age range of 6–30 months (monitored is defined as attending at least one growth monitoring session between July 2019 to August 2020);
•Stable supervisory presence. Local NGOs are contracted to monitor and supervise all community health workers and help to strengthen linkages with local structures such as the community health committees and primary care facilities. Each NGO supervisor is in charge to up to 9 communities, which they visit twice a month. We defined stable presence as not having had NGO turnover within the last year.

### Recruitment

Local agents (the government Regional Nutrition Office and the local NGOs) will carry out the initial recruitment of communities by introducing the ECD curriculum structure and scope. At this time, CHWs (two per community) will indicate whether they would be willing to participate in a two-week training session and implement the ECD group sessions in their community for a one-year period and participate in monthly surveys. If a CHW declines to participate, a replacement community will be chosen. Informed consent for the administration of the survey will be collected by the local agents after this introduction to the planned activities and will be collected again more formally by the survey firm at baseline, after the training has occurred.

### Study arms

#### Control: status quo health and nutrition program

The standard of care program is a community-based health and nutrition program managed by U-PNNC (Unité—Programme de Nutrition Communautaire). The program has a package of activities that address maternal and child health and nutrition (MCH&N) issues:

•Health: child health monitoring, integrated management of non-severe child illness, pregnancy monitoring
•Nutrition: growth promotion, breastfeeding promotion and complementary feeding education, monitoring of pregnant and lactating women, oral rehydration therapy for treatment of severe diarrhea and management of moderate and acute malnutrition

Activities are carried out at the community level by two CHWs, who are elected by the community.

#### Treatment (T): Status quo health and nutrition program + Fortnightly group ECD sessions, based on Reach Up and Learn curriculum and behavioral supporting nudges

##### Community mobilization/information campaign

At the onset of the program, the ECD activities will be presented to the treatment communities during community mobilization campaigns by the program supervisors and the CHWs, supported by local authorities. The mobilization will start with an introduction that raises awareness about the importance of ECD and explains the objectives of the program. It will be followed by a description of the eligibility, enrollment, structure/content of the sessions, and time commitment by the caregiver-child dyads. The dissemination will be supported by photos and videos of the sessions carried out during pre-tests, as well as supporting materials used during the sessions. We may consider selecting individuals within each neighborhood who would be best placed to spread information about the intervention to encourage adherence and elicit possible interest in enrollment.

##### Household recruitment

During these mobilization campaigns, NGO supervisors will collect names from all eligible households who are interested in participating in the ECD sessions. To be eligible for the ECD curriculum, households must have a child between 6–30 months, have a primary caregiver that is willing to participate in the group activities and live in the selected community. Households or caregiver-child dyads could also register with the CHWs or NGO supervisor during the two weeks following the mobilization campaign.

On average, there are about 100 age-eligible mother–child dyads registered in the health and nutrition program in the target communities, and the interventions will be provided for less than half of this eligible number (maximum 40). If there is more demand than can be accommodated for a given age group at a community, all interested households will be entered into a non-public lottery. The research team will randomly draw 10 households from this list to offer a spot in the ECD program, with the remainder being added to a wait list, and will send the final list to the NGO supervisor. Households who are on the wait list will be informed that they have been added to a wait list due to limited space in the program. If enrolled participants do not attend three consecutive ECD sessions (out of the 12 session planned over a period of six months), randomly selected households from the waitlist will be offered to join. There are no additional screening criteria.

##### ECD group sessions

The program will consist of fortnightly group sessions (one to one and a half hour on average) of 10 caregiver-child dyads per six-month age group, for a total of 12 sessions per age group over a period of six months. With four age groups of 10 children each (from 6 to 30 months old), coverage can reach up to 40 children at any given time. Each session is expected to require half a day of the CHW’s time for preparation, administration, filling out of registries, and clean up.

The Reach Up and Learn ECD stimulation curriculum was adapted to a group setting based on the previous MAHAY experience. A session will start by (i) reviewing the previous sessions activities and themes, with a few questions to reinforce the messages, followed by (ii) a discussion among program participants. The CHW will then introduce a theme and message for the session. (iii) a pledge will then be recited by all caregivers and CHWs, highlighting the importance of the caregiver’s commitment. (iv) Caregivers will be grouped in twos or threes to discuss the stimulation activities they did with their own child since the last session. Children and caregivers will spend in total 20 to 30 min on 3–4 play activities using age-appropriate toys. Then, they will all learn a new song to sing with or to the child. (v) The session will end with feedback on the messaging and the distribution of a reminder sheet with the main messages, play activities, lyrics of the song, a list of activities to do at home before the next session, followed by (vi) a closing song.

The age-appropriate play materials used during the group ECD sessions have been adapted from the Reach Up and Learn program’s list. They have been adapted to the materials available in Madagascar. The production and maintenance of the play materials is carried out by a team of trained volunteer youth groups (scouts) (Tily Eto Madagasikara, TEM) in partnership with U-PNNC.

Behavioral nudges will be incorporated into the ECD group sessions to promote peer support and encourage sustained caregiver-child play in between the fortnightly meetings. These nudges will include a little paper booklet (memory aids) that recap the main lessons of the session with pictures and short messages, the lyrics of the song learned and the activities done during the session, and provide suggestions for small feasible actions that can be taken at home to promote learning and child development. Caregivers are instructed to fill in the check-boxes listing the activities they have completed with their child at home and bring back the memory aid to the subsequent group session to share their progress in pairs. These memory aids complement the ECD sessions through light-touch reminders to play, and they can be used to reinforce concepts in between sessions and teach other family members so they can also put the lessons into practice. At the end of the curriculum, each caregiver-child dyad that attended each session will have a complete set of memory aids that recap all the ECD lessons learned. A recent study in Zambia suggests that low-cost visual reminders in the home about target activities to promote child development and nutrition may have positive effects, particularly for the most vulnerable [22].

##### Transitions across age groups

The study period will cover two cycles of the 12 ECD group sessions. After six months, participants of the first three age groups will be offered to “graduate” to the next age group’s curriculum, and new participants will be added to the youngest age group. At this time, if there are participants who decide not to move to the subsequent age group cycle at the transition point, wait list participants will be randomly offered a spot in the age group.

##### Fidelity

CHWs and their NGO supervisors will receive training before the launch of the interventions. Training will include discussion of notion of child development, the importance of early stimulation, the content of the curriculum, with emphasis of role-play content, and assessments of comprehension. An in-person three-days coaching is planned at the onset of the activities and refresher sessions to reinforce concepts and ensure consistent quality across implementers are planned every 4–6 months. In addition, coaches from the ECD team in Madagascar will be available by phone monthly for monitoring and support, and CHWs will have access to phones for technical support.

The NGO supervisors will administer a monthly group session checklist, adapted from the Reach-Up material to assess the quality of the sessions being carried out. Finally, we will develop a brief tracking tool to be used by CHWs to record any changes in toy/play material inventory (losses, deterioration in quality) over time.

##### Treatment Plus (T +): Status quo health and nutrition program + fortnightly group ECD sessions, based on Reach Up and Learn curriculum and behavioral supportive nudges + individual toy boxes with age-appropriate toys for home-based play

After six months of program operation, half of the ECD communities will be randomly allocated to receive an enhanced play materials and activities package for home use to complement the Reach Up and Learn curriculum (T +) (Fig. 2). To promote sustained play between group sessions and daily practice, participating families will receive age-appropriate toy/books boxes at the start of the 6-month cycle. This toy box is composed of one developmentally-appropriate toy given as a gift to the caregiver-child dyad and kept at home. In addition, caregiver-child dyads will be able to borrow a toy and a book from the ECD group session play activities every two weeks to increase play variety and to practice activities learned with the CHWs. Caregivers will learn how to use the toys to play with their children, with lessons at each meeting providing scaffolding to encourage developmental growth. The 6-month delay in implementing T + increases the power to detect CHW time cost differences from integrating ECD group activities (T) with the health and nutrition program, while maintaining sufficient power to detect differences in take up related to the enhanced play materials (T +) by the caregivers (see section Power Calculations).

### Data collection

There are four primary data sources that will be used to collect outcome and key covariate indicators: administrative census data, CHW phone surveys and shadowing, community surveys, and administrative participation data; each of these are discussed below.

#### Administrative census data

A census of all households with pregnant women and children aged 0–5 years was conducted in January 2021 ahead of study activities by U-PNNC as part of the standard MCH&N activities. The census will be used to a) understand underlying characteristics of the study communities and b) identify households with age-eligible children for participation. Households with age-eligible children were given a unique ID and additional information will be gathered including community location, maternal education, number of children 0–5 years old, number of pregnant women and a measure of household wealth.

#### Monthly CHW surveys and shadowing

Phone surveys will be conducted at baseline and monthly with 150 CHWs (two for each of the 75 study communities) by trained enumerators. In addition to the phone/in person monthly survey, enumerators will record all the activities and time spent by CHW on key work activities, including different aspects of the ECD sessions (if in the treatment arms) during shadowing sessions. Originally planned to be administered every other month, it will start as soon as the COVID-19 restrictions for fieldwork are lifted. The data collected from these shadowing visits are meant to triangulate and validate the self-report time use surveys administered over the phone/in person. The time shadowing will be carried out by a trained enumerator accompanying the CHW during a workday (7am to 6 pm).

#### Community surveys

Surveys will be conducted with two key informants (e.g. mayor, community chief, teachers, doctor, religious leaders, etc.) in each community on a monthly basis to obtain information about key community infrastructure, ongoing programs and events, the frequency and duration of local economic/weather/other shocks, and information on market access, and prices of key commodities. Key informants are identified by the CHWs in collaboration with the supervisors. Data collected from these surveys will be used to control for community-level time-varying socio-economic shocks during the study period that may affect our key outcomes of interest.

#### Administrative monitoring data

The main data source for caregiver-child dyad participation and engagement at the community health center will be administrative data from a digital tool used to monitor health and nutrition program activities (CommCare application) as part of their monitoring information system. Individual data on participation in program activities are collected by the CHWs on a paper monitoring registry, as well as an ECD-specific monthly monitoring registry. The NGO supervisors during one of the monthly visits to the community, enters the individual participation data contained in the registries into the CommCare application using the unique caregiver-dyad identification number provided by the census.

### Outcomes

The following primary and secondary outcomes will be collected:

•*CHW time allocation* (Primary outcome), measured through a 24-h recall of time spent on program related tasks (group nutrition sessions, home visits, treatment of illnesses, administrative tasks, ECD group sessions, and toy maintenance), household chores, productive activities, caregiving and rest/social activities. This will be measured at baseline and monthly for 12 months through phone-surveys.
•*CHW psychosocial wellbeing* (Secondary outcome), measured through the monthly phone surveys. Motivation and depression will be measured at baseline and every four months using the short-form Center for Epidemological Studies Depression Scale (CES-D) [23] and motivation scales [24] adapted to the Malagasy context and the Rosenberg Self-Esteem Scale [25]. Perceived stress will be measured monthly using the Perceived Stress 7-Item Scale (PSS-7) [26].
•*Caregiver-child dyad participation in health and nutrition activities* (Primary outcome), measured through the administrative data monthly. The range of program activities that are collected range from monthly attendance to group promotion sessions and growth monitoring sessions, indicators of whether they received home visit in last month, and whether they received vitamin A supplementation, deworming and vaccinations (age-specific).
•*Caregiver-child dyad participation in ECD sessions* (Primary outcome), measured through the administrative data monthly. Information ranges from number of ECD sessions attended by caregiver-child dyad in last month and whether the children transitioned from one age group to the subsequent one.

### Power calculations

#### CHW time allocation

We assumed repeated observations (baseline and 11 post implementation), with an auto-correlation coefficient of 0.7 (high) or 0.5 (low) for the correlation between follow-up measurements of CHW time allocation. We will control for baseline outcomes with an ANCOVA specification. We adjusted for the clustering design effect on sample size with an adjustment of (1 + ρ(m—1)), where ρ is the intra-cluster correlation (ICC) and m = 2 CHW within community or cluster. We assume an ICC of 0.2 and 0.3 on the activities performed by CHWs within a cluster, where an ICC = 0.2 is benchmarked on previous MAHAY CHW outcomes [27].

Using Stata Corp sampsi command, we performed sample size calculations with 80% power for an expected effect size of 0.5 SD in a bivariate comparison. A conservative sample size is 25 clusters per study arm (Table 1).

**Table 1:** Sample size calculations: number of clusters per study arm

	Autocorrelation coefficient	
	High (0.7)	Low (0.5)
ICC = 0	15	19
ICC = 0.2	18	23
ICC = 0.3	20	25

#### Caregiver-child dyad participation/take up

Assuming participation comparisons between a minimum of 25 treatment and 25 control communities (50 clusters), we modeled the minimum detectable effect (MDE) size using the software package, Optimal Design (OD) Plus Version 3.01, for clustered randomized trials with repeated measures. We will have administrative data for 40 eligible caregiver-child dyads per community participating in the MCH&N programs at each of 12 time points. We assume an ICC ranging from 0.1 to 0.3 for participation of caregiver-child dyads in activities. Under these assumptions, we are powered for MDEs ranging between 0.31 SD (ICC = 0.1) and 0.47 SD (ICC = 0.3).

### Sampling, stratification and randomization

In order to capture efficiency gains from adjusting from observable differences in the design of the randomized experiment, we conducted stratified sampling [28]. We partitioned the covariate space into 16 strata, with stratification criteria that included region (Amoron’i Mania and Haute Matsiatra), remoteness of the community (above/below the median Euclidean distance to the closest health center), population size of the catchment area (above/below median number of children in the 6–30 months old age range), and CHW education level (above/below secondary level of educational attainment).

Within each of the 16 strata, we sampled six or 12 communities (one sextuplet drawn from each of seven smaller strata and two sextuplets drawn from each of nine large strata) to create a set of 25 sextuplets that are comparable along the stratification dimensions. Next, we randomly drew three communities selected from each sextuplet and allocated each to one of three possible arms (i.e. allocation ration of 1:1:1 in two treatment arms and one control arm) for a total of 75 communities in the final sample. This sampling approach ensures that each community has a cluster of comparable communities within its assigned stratum and allows for up to three back up communities for the potential need for replacement in case of refusal or drop-out by participating communities [28, 29]. All sampling and randomization were done by key research personnel using standard and reproducible computer-assisted software packages in Stata 16.

The random generation sequence and the random assignment of (i) communities to treatment arms and of (ii) households in case of over-subscription in T and T + will be performed by the research team. Due to the nature of the interventions, it is impossible to blind participants to their intervention group assignment. Interviewers will not be blinded as they will ask participants about their program-related experiences. However, the data will be masked to group assignment during the data analysis phase to blind the data analysts.

### Data management

Data collection will be conducted by the National Statistical Institute (INSTAT), which has extensive experience in collecting household survey data in Madagascar. We will collect data with Computer Assisted Personalized Interviews (CAPI) on Android tablets using Survey Solutions, a free software platform.

For both survey and administrative data, subject key (identifiers and subject numbers) will be kept in a secure separate location. Individual-level data collected from the census and administrative sources will be shared with the research team for the purposes of analysis with each individual having a unique respondent ID code. All documentation regarding the participants, including the consent forms and questionnaires, will be identified using appropriate participant codes. All records will be accessible only to approved survey firm personnel and research team members in password-protected files. Researchers will also make no attempts to identify any individual caregivers or children through a combination of any of the variables provided through this administrative data. In the event of a breach of confidentiality, we also consider the potential harm to subjects to be minimal as we are not collecting sensitive data.

### Statistical methods for primary and secondary outcomes

#### General analytic approach

Primary analyses will be conducted on an intention-to-treat (ITT) basis.

Identification of causal impacts will rely on the random assignment of treatment at the community level. The main impact of the program activities will be measured through a series of pair-wise comparisons of mean outcomes between the study arms. However, it is possible that by chance alone we will find a covariate imbalance between communities in different treatment arms that could confound our estimates. Therefore, we will measure theoretically significant covariates at the caregiver-child dyad level, CHW and community levels to include in a regression framework.

The potential for contamination or spillover effects for CHWs and caregiver-child dyads across communities for the program is low, as communities are self-contained and provide services to mothers and children registered in the local community.

#### Parameters of interest

Here, we describe the parameters of interest and introduce notation. Treatment, randomly assigned at the community level, is represented by two arms: group ECD sessions (T), and group ECD sessions + enhanced play materials (T +). The status quo, or control arm, is denoted by C. In notation terms, the full set of potential assignment variants is given by t = [C, T, T +] A set of individual (CHW and/or caregiver-child dyad, depending on the outcome) level covariates, X, and community-level covariates, V, will be used to adjust for imbalance that may occur by chance in the randomization process. We also include the baseline value of the outcome of interest (Y_0_) to improve precision in our models. Below, we report the parameters of interest for the primary and secondary research questions (reproduced below). Parameters that differ in outcome but not in notation by research question are combined for simplicity.

- *Primary Question 1: How does the integration of ECD activities into existing health and nutrition responsibilities affect the time use and task allocation of CHWs?*
- *Secondary Question 1: How does the integration of ECD activities affect measures of CHW psychosocial wellbeing?*
- *Primary Question 2: How does the integration of ECD activities affect participation by caregiver-child dyads in all activities led by CHWs?*

The estimands are pair-wise comparisons of each intervention arm with the control arm, for CHW or caregiver-child dyad outcomes. These comparisons are represented by the following equation:

1 $$\mathrm E\left[\mathrm Y\left|D=d,Y_0,X,V\rbrack-E\lbrack Y\left|C,Y_0\right.\right.,X,V\right],where\;d=\lbrack T,T+\rbrack$$

- *Primary Question 3: How does the availability of age-appropriate materials/activities for home-based play affect caregiver-child dyad participation in ECD activities?*

To understand how integration of toy boxes affects time/task allocation of CHWs or caregiver-child participation, we will do one pairwise comparison between the two intervention arms for this outcome. This comparison is represented by the equation below:

2 $$\mathrm E\lbrack\mathrm Y\vert\mathrm D=\mathrm T,{\mathrm Y}_0,\mathrm X,\mathrm V\rbrack-\mathrm E\lbrack\mathrm Y\vert\mathrm D=\mathrm T+,{\mathrm Y}_0,\mathrm X,\mathrm V\rbrack$$

- *Secondary Question 2: How is the treatment effect on caregiver-child dyad participation in health and nutrition activities moderated by key household and community characteristics?*

In order to estimate how the treatment effect obtained in Eq. (1) for caregiver-child dyad participation is moderated by key individual and community-level characteristics, M, we use the following estimand:

3 $$\left(\mathrm E\left[\mathrm Y\vert\mathrm D=\mathrm d,{\mathrm Y}_0,\mathrm X,\mathrm V,\mathrm M=1\right]-\mathrm E\left[\mathrm Y\vert\mathrm C,{\mathrm Y}_0,\mathrm X,\mathrm V,\mathrm M=1\right]\right)-(\mathrm E\left[\mathrm Y\vert\mathrm D=\mathrm d,{\mathrm Y}_0,\mathrm X,\mathrm V,\mathrm M=0\right]-\mathrm E\lbrack\mathrm Y\vert\mathrm C,{\mathrm Y}_0,\mathrm X,\mathrm V,\mathrm M=0\rbrack),\mathrm{where}\;\mathrm d=\lbrack\mathrm T,\mathrm T+\rbrack$$

#### Empirical specification

We have two primary estimating equations, which we discuss below. The first (Eq. 4) is our main specification for comparisons between each intervention arm and the control group (Primary Questions 1 & 2, Secondary Question 1). These models will be used to estimate the impact on CHW outcomes (time use and motivation) and caregiver-child dyad outcomes (participation and engagement). All specifications will control for the method of randomization by including strata indicator variables, month fixed effects to account for time-varying unobservable (to the researcher) factors, and correcting standard errors for clustering at the community level. For clarity, we reproduce one set of equations here, but they can be applied to either CHW-level or caregiver-child dyad-level analysis.

Let the outcomes of interest will be represented by ${Y}_{ijt}$, where the subscript refers to CHW (or caregiver-child dyad) *i*, in community *j*, at time *t*.

The main ITT impact of each intervention arm on the outcome of interest is estimated as follows:

4 $${Y}_{ijt} = \alpha + {\beta }_{1}{T}_{j} +{\beta }_{2} {{T}_{+}}_{j}+ \gamma {Y}_{ij0} + {\delta }_{1}{X}_{ijt}+ {\delta }_{2}{V}_{jt}+ {\theta }_{s}+ {\lambda }_{t} + {\varepsilon }_{ijt}$$

where ${T}_{j}$ or ${{T}_{+}}_{j}$ are indicators for the random assignment of community j to treatment, ${Y}_{ij0}$ are baseline outcomes for CHW (or caregiver-child dyad) i (ANCOVA specification), ${X}_{ijt}$ are CHW-level (or caregiver-child level) covariates (e.g. age, gender, education level), are ${V}_{jt}$ are time-varying community-level characteristics (e.g. shocks, prices/wages), ${\theta }_{s}$ are strata fixed effects (randomization triplet fixed effects), ${\lambda }_{t}$ is a set of month fixed-effects, and ${\varepsilon }_{ijt}$ are error terms clustered at the community level.

β_1_ and β_2_ represent the average impact of being assigned to T or T + over 12- and 6-month periods, respectively, relative to the control group. Testing primary question 1 and primary question 2 is equivalent to testing whether (H0: β_1_ = 0 and (H0: β_2_ = 0).

To address Primary Question 3, we restrict the sample to the communities that are offered any ECD intervention (T or T +) and estimate the following equation:

5 $${Y}_{ijt} = \alpha + {\beta }_{2} {{T}_{+}}_{j}+ \gamma {Y}_{ij0} + {\delta }_{1}{X}_{ijt}+ {\delta }_{2}{V}_{jt}+ {\theta }_{s}+ {\lambda }_{t}+ {\varepsilon }_{ijt}$$

These analyses will occur in a restricted sample of only communities where T = 1 or T + = 1. Here, testing (H0: β_2_ = 0) allows to test whether being assigned T + provides any additional benefit over and above T.

#### Subgroup analyses

For Secondary Question 2, subgroup analysis will be carried out by documenting the differential impact of the two interventions along key household and community-level characteristics. We will estimate heterogeneity on the pooled treatment arms interacting the characteristic of interest with the treatment variable, as well as explore the use of recent developments in machine learning methods [30].

#### Missing data

Multiple imputation by chained equations will be used for missing covariate and baseline outcome information. Sensitivity analyses will be performed with and without imputation [31].

#### Interim analyses

Since the ECD curriculum is designated for a specified time period per age group and the intervention does not present any risk to the enrolled population or beneficiaries of the intervention, there are no formal rules for stopping this trial. Survey and administrative data will be reviewed monthly for assessing quality and completeness. If any adverse event, whether related to the intervention or not is reported to the local NGO or U-PNNC, the research team and relevant regulatory bodies will be notified to examine the severity or seriousness of the event.

### Provisions for post-trial care

There are no anticipated direct benefits or harms for participation in the trial or after the trial has ended. All caregivers that participated in the session, as well as other community members, will continue to have access to receive the benefits of the government nutrition program (standard of care provided to the control arm), and the treatment arm will continue to receive standard of care after the intervention has ended. Incorporation of results from the current study will feed back into the redesign of the integrated package by U-PNNC to be further tested or scaled up in the next phase of the project.

We used the SPIRIT checklist to design the structure of the protocol. [30] The results of this study will be submitted to peer-reviewed journals.

## Discussion

The overall objective of the study is to examine the effects of integrating ECD group sessions into the existing, at-scale, community health and nutrition programs administered by the government in Madagascar. To achieve these objectives, we will use a cluster randomized controlled trial design with three arms: a control group, with the status quo, a community-based health and nutrition program; a treatment arm with the addition of fortnightly group ECD sessions, based on Reach Up and Learn curriculum and supportive behavioral nudges, to the status quo; and an enhanced treatment arm, where the intervention in the treatment arm is layered with the provision of individual toy boxes including age-appropriate toys. The results from the trial will provide the evidence base required to implement an integrated package of nutrition, health and ECD promotion activities at scale in Madagascar, and findings may be relevant in other low-income countries.

### Trial status

Data collection is ongoing and will conclude in March 2022.

## Data Availability

In accordance with the World Bank’s Open Data and Knowledge Initiative, the deidentified data collected in the study will be made publicly available at the data repository at the World Bank, which is expected to be within 24 months of the final data collection date. Metadata and critical documents (i.e. protocols and questionnaires) will conform to the standards of the Data Documentation Initiative and will be made available within one year of the end of data collection.

## Abbreviations

ONN: L'Office National de Nutrition
U-PNNC: Unité: Programme de Nutrition Communautaire
MCH&N: Maternal Child Heath & Nutrition
TEM: Tily Eto Madagasikara
INSTAT: National Statistical Institute
